# Vereinbarkeit von Care-Arbeit und Facharztweiterbildung Psychiatrie und Psychotherapie – Chancen besser nutzen: Positionspapier der Generation PSY – Nachwuchsinitiative der Deutschen Gesellschaft für Psychiatrie und Psychotherapie, Psychosomatik und Nervenheilkunde e. V. (DGGPN)

**DOI:** 10.1007/s00115-024-01793-4

**Published:** 2025-01-20

**Authors:** Nina Schubotz, Linda Meyer, Katrin Radenbach

**Affiliations:** 1 Alexianer St. Joseph-Krankenhaus Berlin-Weißensee, Gartenstraße 1, 13088 Berlin, Deutschland; 2 Alexius/Josef Krankenhaus, Nordkanalallee 99, 41464 Neuss, Deutschland; 3 Klinik für Alterspsychiatrie, Ökumenisches Hainich Klinikum gGmbH, Pfafferode 102, 99974 Mühlhausen, Deutschland

Bei vielen Ärzt:innen überschneiden sich Familiengründung und ärztliche Weiterbildung. Vor dem Hintergrund des ärztlichen Nachwuchsmangels [7], der nicht zuletzt auch die Psychiatrie betrifft, ist die Verbesserung der Vereinbarkeit von Care-Arbeit und beruflicher Tätigkeit im Ärzt:innenberuf von großer Dringlichkeit. Unter Care-Arbeit oder Sorgearbeit verstehen wir dabei jegliche Aktivitäten der Kinderbetreuung sowie Pflege und Betreuung hilfsbedürftiger Angehöriger. Verpflichtungen durch die Pflege Angehöriger werden vor dem Hintergrund der demographischen Entwicklung weiter zunehmen. Zudem schätzen Ärzt:innen die Vereinbarkeit ihrer beruflichen Tätigkeit mit privaten Interessen von Generation zu Generation wichtiger ein [9]. Aufgrund der fortbestehenden Stigmatisierung psychischer Erkrankungen und psychiatrischer Institutionen steht der Fachbereich vor zusätzlichen Herausforderungen bei der Nachwuchsgewinnung.

Das Positionspapier des Bündnis Junge Ärzte [1] formulierte bereits 2016 fächerübergreifend klare Forderungen an Politik, Ärztekammern und Klinikleitungen zur Verbesserung der Vereinbarkeit von Beruf und Familie. Bislang besteht jedoch weiterhin erhebliches Verbesserungspotenzial [2, 9, 10].

Die Psychiatrie bietet hinsichtlich der allgemeinen Arbeitsbedingungen Vorteile im Vergleich zu anderen Fachbereichen. Im Jahr 2023 hat die Deutsche Gesellschaft für Psychiatrie und Psychotherapie, Psychosomatik und Nervenheilkunde e.V. (DGPPN) eine zweimonatige Online-Weiterbildungsevaluation (Manuskript angenommen bei *Der Nervenarzt*, im Publikationsprozess) mit 315 Ärzt:innen in Weiterbildung Psychiatrie und Psychotherapie sowie Fachärzt:innen mit einem Facharzttitel seit weniger als 3 Jahren durchgeführt. Die Ergebnisse zeigen im Vergleich zur fächerübergreifenden Hartmannbund-Studie von 2021 mit 1258 Ärzt:innen in Weiterbildung [6], dass Ärzt:innen in Psychiatrie und Psychotherapie weniger Überstunden machen und häufiger in Teilzeit tätig sind. Die Facharztweiterbildung Psychiatrie und Psychotherapie beinhaltet jedoch auch einige besondere Herausforderungen. Die spezifische psychotherapeutische Qualifikation ist unerlässlich für ein qualitativ hochwertiges psychiatrisches Arbeiten und verpflichtender Bestandteil der Facharztweiterbildung. Die aktuelle Umsetzung birgt jedoch vielerorts besondere Schwierigkeiten für Eltern. So müssen viele Ārzt:innen in Weiterbildung die 600 h Theorie, Selbsterfahrung, Fallbesprechungen, Supervision und Psychotherapiestunden [4] teilweise eigenständig organisieren und finanzieren.

Die Weiterbildungsevaluation der DGPPN zeigte, dass nur 11 % alle verpflichtenden Veranstaltungen in ihrer Weiterbildungseinrichtung absolvieren können. 71 % der Befragten müssen zudem verpflichtende Weiterbildungsteile (Theoriestunden, Selbsterfahrung, Supervision etc.) privat finanzieren. Die vorgeschriebenen Psychotherapiestunden können nur von 60 % vollständig und von 25 % teilweise an der Weiterbildungseinrichtung absolviert werden. Überstunden für externe Weiterbildungsveranstaltungen außerhalb der regulären Arbeitszeit sind häufig, werden jedoch bei 84 % nicht vergütet. Die zeitliche und finanzielle Belastung durch externe Weiterbildungsveranstaltungen, zusätzlich zu in der Regel starren Arbeitszeiten, Bereitschaftsdiensten und natürlich auch in diesem Fachgebiet vorkommenden Überstunden, sind insbesondere für Eltern schwer realisierbar. Häufig bleibt trotz Teilzeitstelle wenig Zeit für private Verpflichtungen. Zudem sind bei 32 % der Befragten nicht alle verpflichtenden Rotationen in Teilzeit möglich. Außerdem zeigte sich in der Weiterbildungsevaluation ein Mangel an Transparenz und Planbarkeit bei der Vergabe von Rotationen. Die Planbarkeit ist jedoch insbesondere für Personen mit zusätzlichen Verpflichtungen in der Care-Arbeit von großer Bedeutung.

Ärzt:innen können, wie alle anderen Arbeitnehmer:innen, nach Antrag jederzeit befristet oder unbefristet in Teilzeit arbeiten (§ 8 Abs. 1 TzBfG). Laut Hartmannbund-Studie von 2021 [6] wünschen sich 56 % der Befragten eine Reduktion der Arbeitszeit. Die Auswirkungen einer Teilzeittätigkeit auf Aufstiegschancen, Dauer und Qualität der Facharztweiterbildung (u. a. schlechterer Zugang zu Rotationen und für die Weiterbildung relevante Inhalte, fehlende Teilnahmemöglichkeiten an Lehrveranstaltungen etc.) sind jedoch bedeutsam [6]. Die Weiterbildungsordnung legt zudem eine minimale Tätigkeit von 50 % fest. Bei manchen Landesärztekammern ist eine Weiterbildung in Teilzeit auch nur auf Antrag möglich. An Universitätskliniken sind Verpflichtungen aus der Patient:innenversorgung, Lehre und Forschung ohnehin schwer zu vereinbaren [3, 8]. Die häufig kurzen Vertragslaufzeiten [9] erschweren zusätzlich eine langfristige Lebensplanung. Außerdem wurde in der Weiterbildungsevaluation deutlich, dass 38 % der Befragten Vorbilder für die Vereinbarkeit von Familie und Beruf an ihrem Arbeitsplatz fehlen.

Der Frauenanteil in der Psychiatrie und Psychotherapie ist hoch, sodass Konzepte für die Gestaltung des Arbeitsplatzes für Schwangere wichtig sind. Die Sicherheit am Arbeitsplatz ist zentral. Gleichzeitig sollte eine Fortführung der ärztlichen Tätigkeit während der Schwangerschaft in für die Weiterbildung relevanten und anrechenbaren Bereichen ermöglicht werden.

Die Chancen der Digitalisierung werden bislang in allen Fachbereichen nur unzureichend genutzt [6]. Eine flächendeckende Digitalisierung der ärztlichen Dokumentation und die Vermeidung von Doppeldokumentation werden von jungen Ärzt:innen jedoch als wichtig erachtet [6], reduzieren die Arbeitsbelastung und Überstunden, ermöglichen die Flexibilisierung der Arbeitszeiten und erhöhen die Behandlungssicherheit und -qualität. Viele ärztliche Tätigkeiten wie die Erstellung von Arztbriefen und ärztlichen Stellungnahmen, Anforderungen, Anordnungen etc. sind bei vollständiger Digitalisierung ortsunabhängig möglich und bieten bei der Ausführung häufig eine gewisse zeitliche Flexibilität. Mobile Arbeitsformen und flexiblere Arbeitszeiten durch beispielsweise Durchführung spezifischer Tätigkeiten außerhalb der Kernarbeitszeiten im Home Office kommen Ärzt:innen mit familiären Verpflichtungen entgegen. In der Weiterbildungsevaluation der DGPPN von 2023 zeigte sich jedoch, dass flexible Arbeitszeiten bislang nur bei 29 % möglich sind, mobiles Arbeiten lediglich bei 11 % der Befragten.

Flächendeckend muss eine transparente und strukturierte Weiterbildung geschaffen werden, die planbare Rotationen mit Teilzeitoption und eine kostenneutrale, in die regulären Arbeitszeiten integrierte Psychotherapieweiterbildung auf hohem qualitativem Niveau beinhaltet. Dafür ist eine klare Regelung der Finanzierung der ärztlichen Weiterbildung unerlässlich [5]. Nur so kann dem eklatanten Nachwuchsmangel entgegengewirkt und eine qualitativ hochwertige Patient:innenversorgung sichergestellt werden sowie die Psychiatrie und Psychotherapie zukunftsfähig bleiben.

Die Generation PSY ist ein ehrenamtlicher Zusammenschluss junger Ärzt:innen in Weiterbildung und Fachärzt:innen in Psychiatrie und Psychotherapie, die sich als Arbeitsgruppe der DGPPN dem Einbringen der Perspektive der jüngeren Generation in die Themen Bekämpfung des Nachwuchsmangels und Entstigmatisierung psychischer Erkrankungen sowie der kritischen Reflektion der aktuellen Arbeits- und Weiterbildungsbedingungen widmet. Diese Arbeit beinhaltet u. a. die Mitgestaltung des Nachwuchsprogramms auf dem jährlichen DGPPN-Kongress sowie der Summer Schools und die Organisation eines Mentoringprogramms für interessierte Studierende und junge Ärzt:innen.
